# Supplementary figures and images for: A conserved microtubule-binding region in Xanthomonas XopL is indispensable for induced plant cell death reactions
Source: PLoS Pathog. 2023 Aug 14;19(8):e1011263. doi: 10.1371/journal.ppat.1011263 (PMC10449215; doi:10.1371/journal.ppat.1011263)

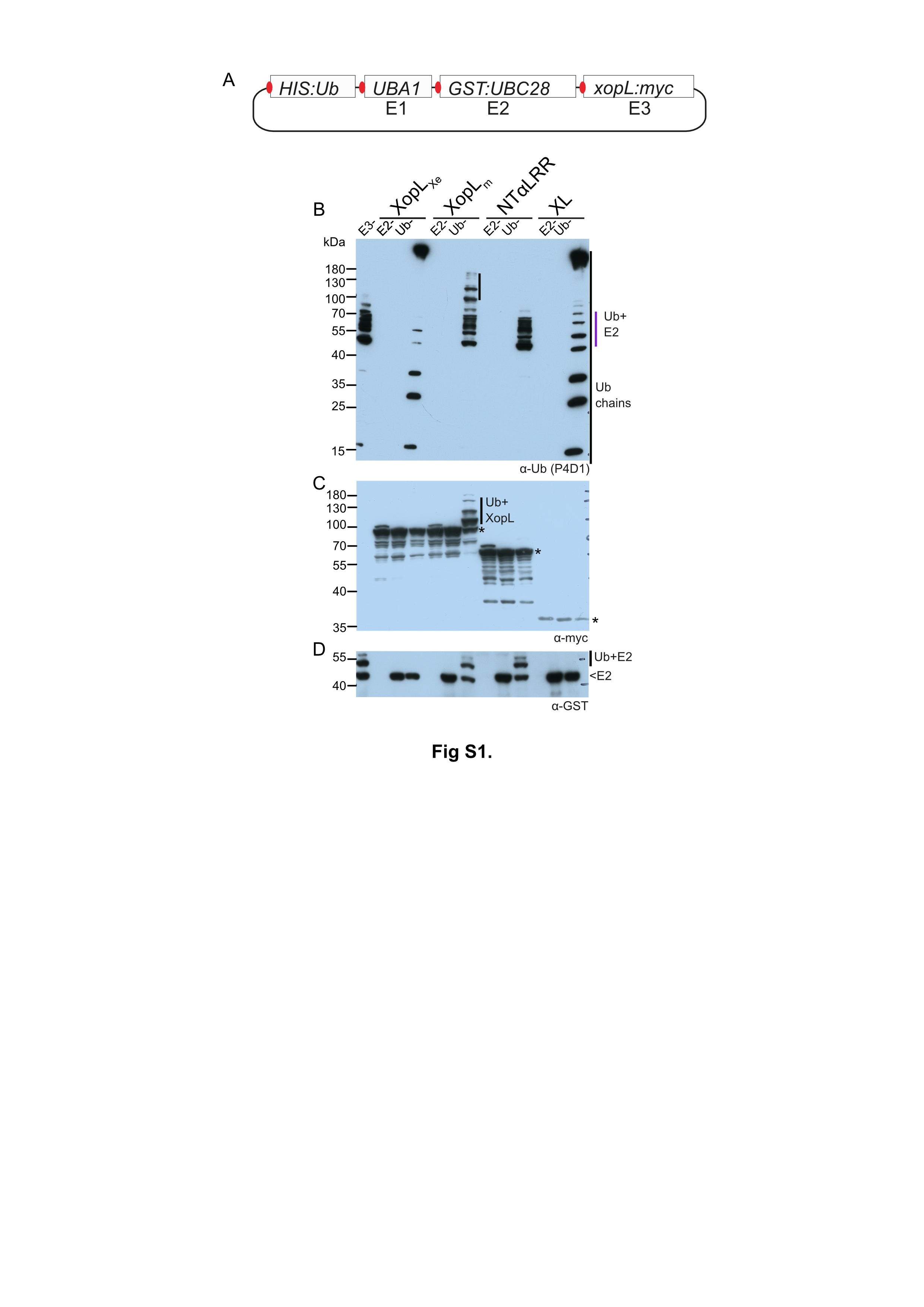

Supplement: S1 Fig — [12]. (A) Schematic of the UbiGate plasmid used to test for E3 ligase activity of XopL proteins. Components of the A. thaliana ubiquitination machinery (affinity-tagged for detection by western blot), UBIQUITIN 10 (HIS:Ub), UBIQUITIN ACTIVATING ENZYME 1 (UBA1) and UBIQUITIN CONJUGATING ENZYME 28 (GST:UBC28) were expressed from a single, IPTG-inducible plasmid together with XopL coding sequences. Each translational unit was equipped with an independent ribosome binding site (red). (B-D) Western blot analysis of protein extracts isolated from E. coli expressing different XopL variants. Controls were samples lacking the E3 ligase (E3-), the E2 enzyme (E2-) or ubiquitin (Ub). The XopL protein variant tested is indicated above the designated lanes. (B) Ubiquitin was detected using the P4D1 antibody. Polyubiquitin chains are indicated by a black line. E2-ubiquitin (Ub+E2) conjugates are visible in the E3- control sample, XopLm and NTαLRR samples (size indicated by a purple line). (C) C-terminally tagged XopL proteins were detected with a myc-specific antibody (expected size indicated with ‘*’). In some cases, autoubiquitination is detectable (Ub+XopL). (D) N-terminally-tagged E2 enzyme was detected using a GST-specific antibody (expected size indicated with ‘*’). E2-ubiquitin conjugates are indicated with a black line. Samples were run on different gels to clearly visualize ubiquitin and other proteins. Protein mass is expressed in kDa. (TIFF) [file ppat.1011263.s001.tiff]

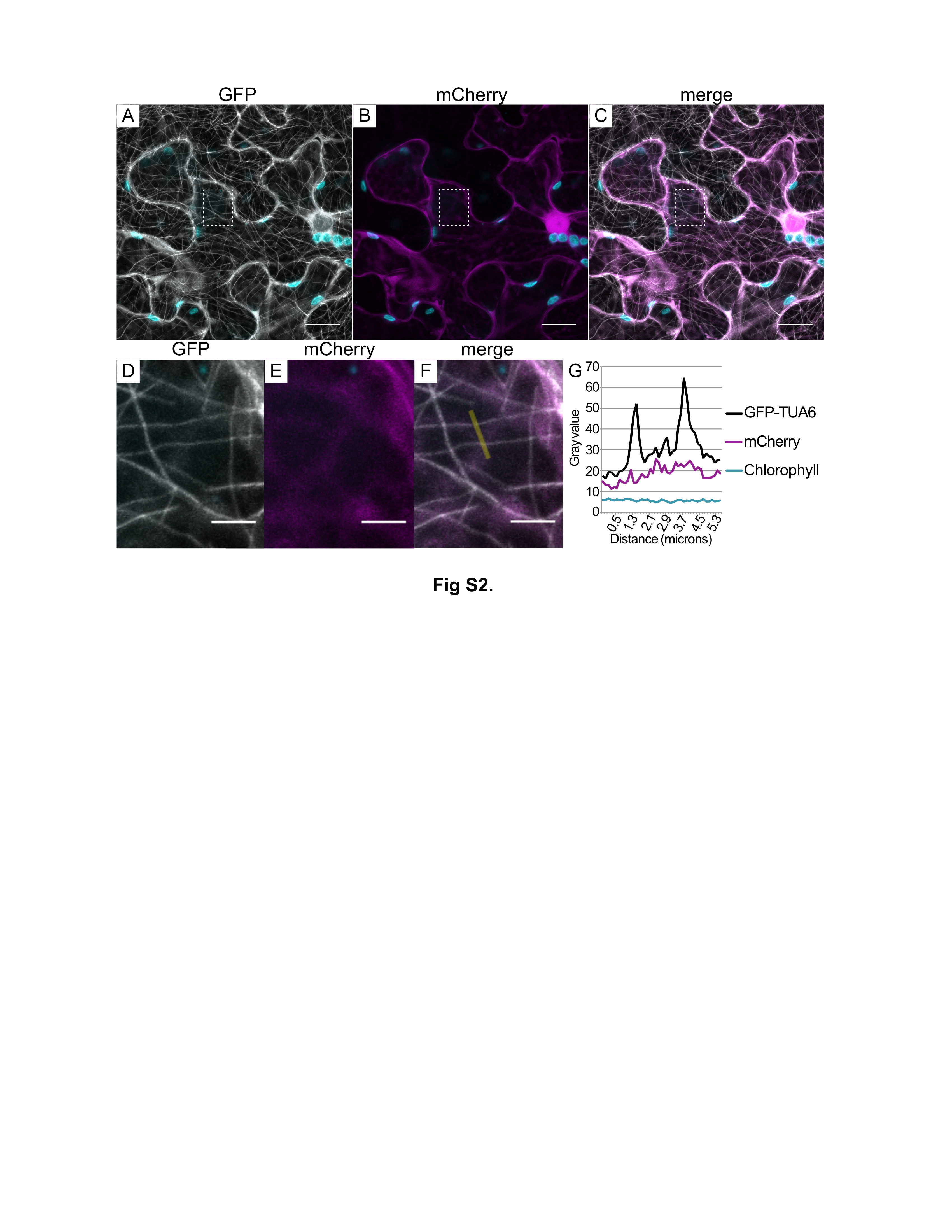

Supplement: S2 Fig — Confocal microscopy of a lower epidermal cell of a GFP-TUA6 (labels MTs) transgenic N. benthamiana leaf. The leaf was agroinfected to express mCherry (OD600 of 0.4). Samples were harvested for microscopy 2 dpi. (A) GFP-labeled microtubules are in white, (B) mCherry in magenta, (C) is a merged image of (A) and (B). Plastids are colored in cyan. Scale bars are 20 μm. (D-F) are magnified from images (A), (B) and (C), respectively. Scale bars are 5 μm. The yellow line in (F) shows the location of the intensity plot measurement depicted in (G). (G) Fluorescence intensity plot across two MTs (visible as two distinct peaks in black). mCherry and chlorophyll intensity profiles were included as negative controls. (TIFF) [file ppat.1011263.s002.tiff]

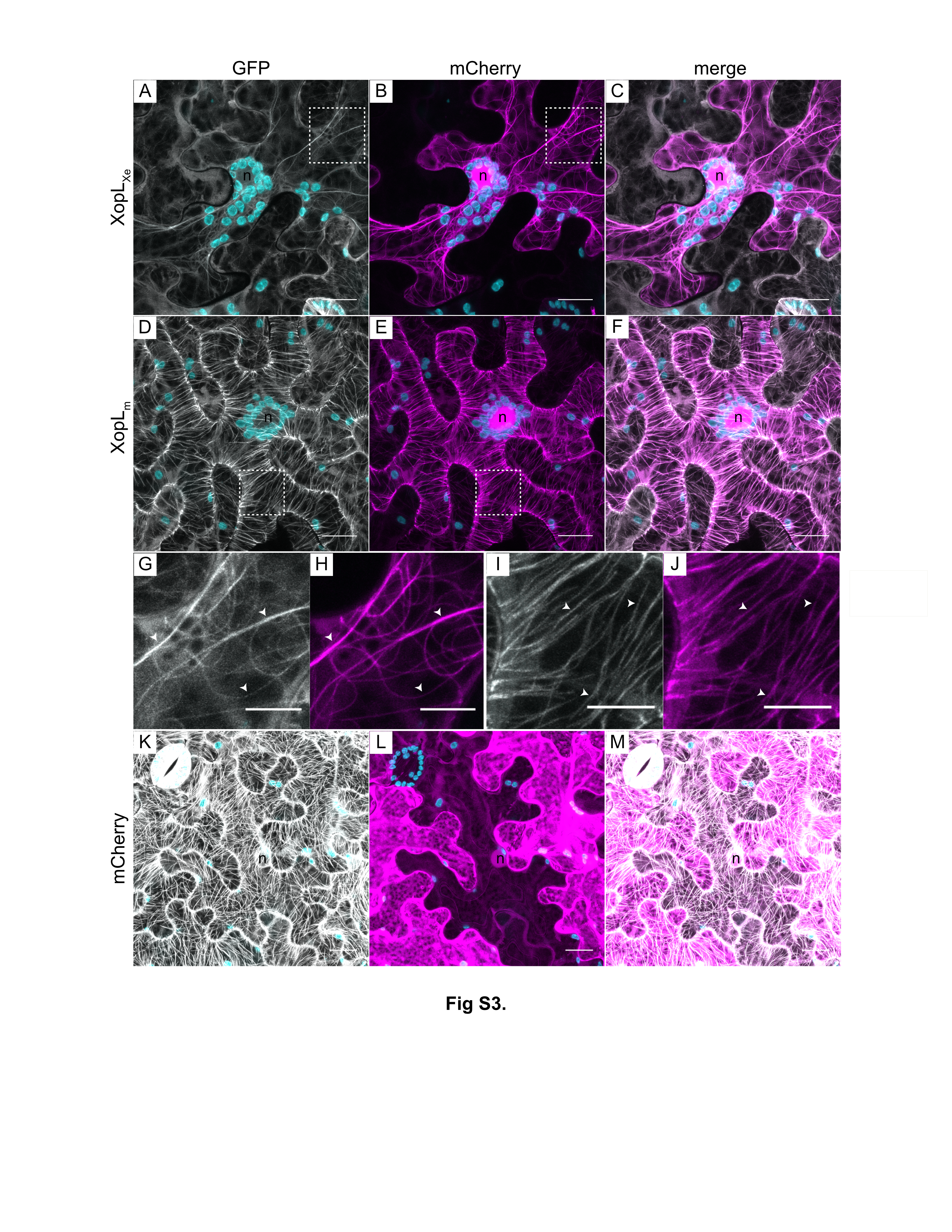

Supplement: S3 Fig — Confocal microscopy of lower epidermal cells of GFP-TUA6 (labels MTs) stable transgenic N. benthamiana leaves. Leaves were agroinfected (OD600 of 0.4) to express (A-C) XopLXe-mCherry, (D-F) XopLm-mCherry and (K-M) mCherry and treated with the MT stabilizing chemical taxol (solved in DMSO) at 4 hpi. Samples were harvested for microscopy 2 dpi. The GFP channel is visible in white (labeled MTs) and the mCherry channel in magenta. Plastids are in cyan; ‘n’ labels nuclei. Scale bars are 20 μm. (G), (H), (I), (J) Are magnified images from (A), (B), (D) and (E), respectively (area magnified is outline with a white box). Scale bars in (G-J) are 5 μm. Examples of MTs are labeled with white arrows. (TIFF) [file ppat.1011263.s003.tiff]

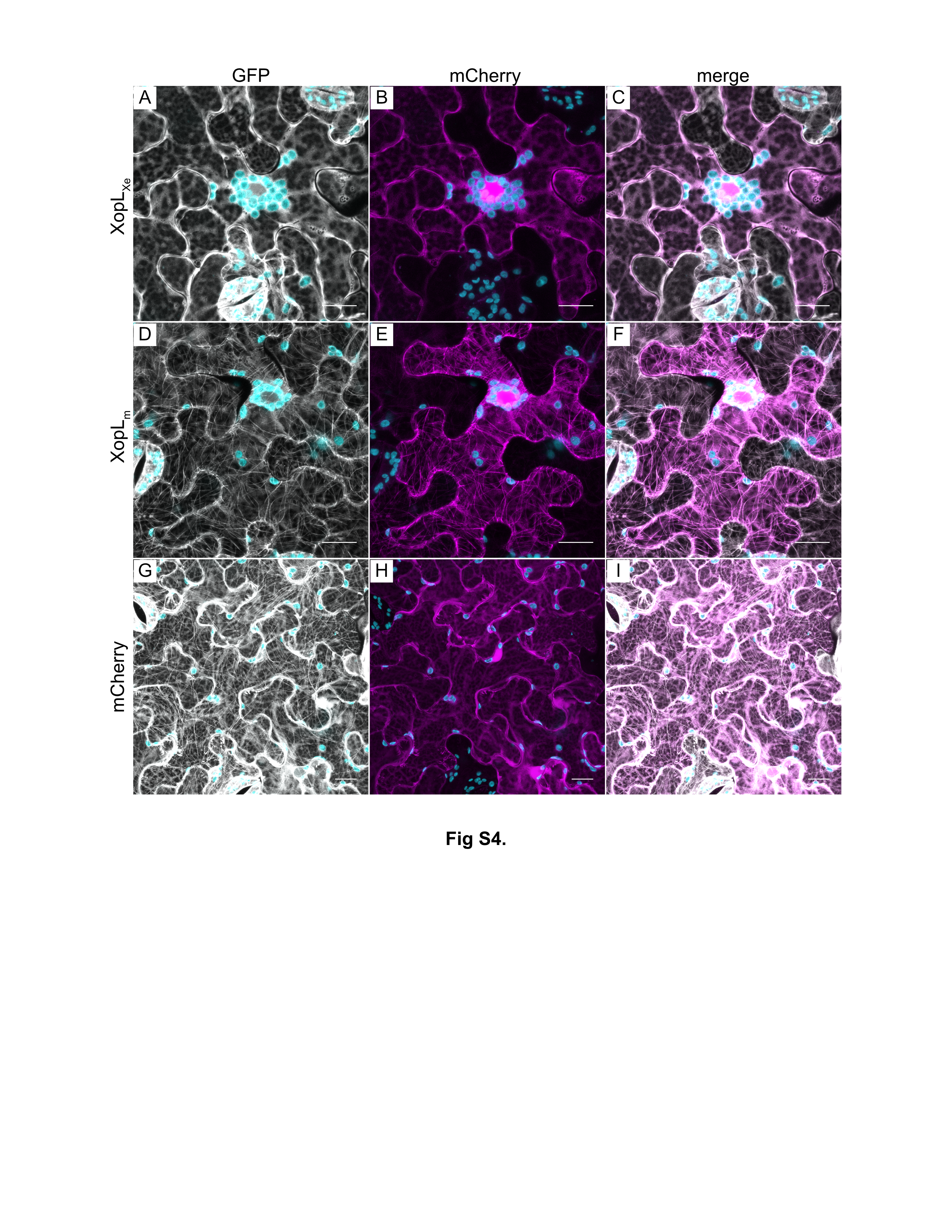

Supplement: S4 Fig — Confocal microscopy of lower epidermal cells of GFP-TUA6 (labels MTs) stable transgenic N. benthamiana leaves. Leaves were agroinfected (OD600 of 0.4) to express (A-C) XopLXe-mCherry, (D-F) XopLm-mCherry and (K-M) mCherry which was then treated with DMSO at 4 hpi. Samples were harvested for microscopy 2 dpi. The GFP channel is visible in white (labeled MTs), the mCherry channel in magenta. Plastids are visible in cyan. Scale bars are 20 μm. (TIFF) [file ppat.1011263.s004.tiff]

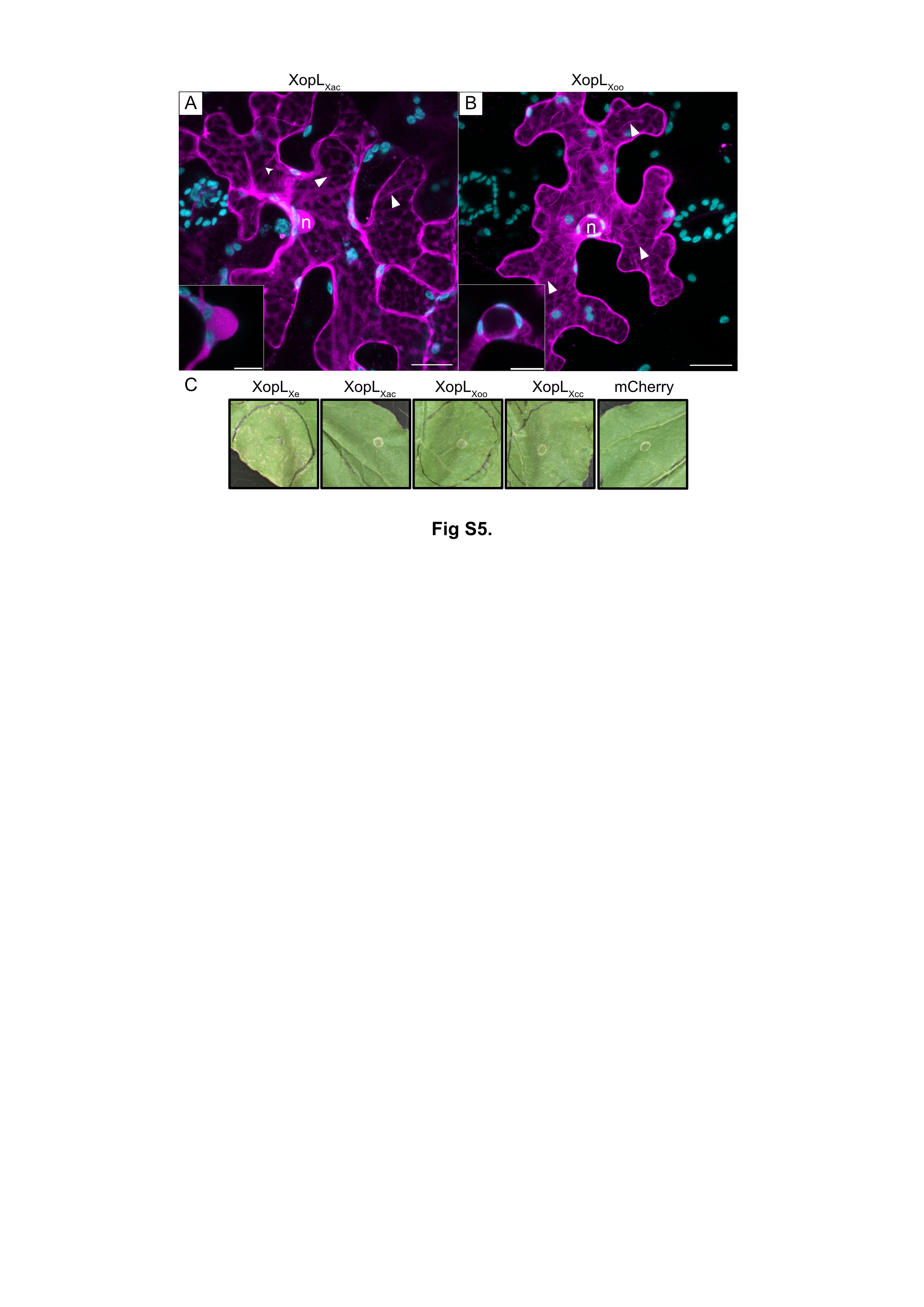

Supplement: S5 Fig — Confocal microscopy of lower epidermal cells of GFP-TUA6 stable transgenic N. benthamiana leaves. Leaves were agroinfected (OD600 of 0.4) to express synthesized (codon-optimized) (A) XopLXac and (B) XopLXoo translationally fused to a C-terminal mCherry. (A) and (B) are zoomed-out versions of cells depicted in Fig 4G and Fig 4J, respectively. mCherry-tagged XopLs are visible in magenta and the GFP channel is not shown here, ‘n’ marks the nucleus, white arrows show example MTs. Scale bars are 20 μm. Insets are magnifications of the nuclei (scale bar is 10 μm). (C) Plant reactions to codon-optimized XopLs were monitored at 6 dpi. (TIFF) [file ppat.1011263.s005.tiff]

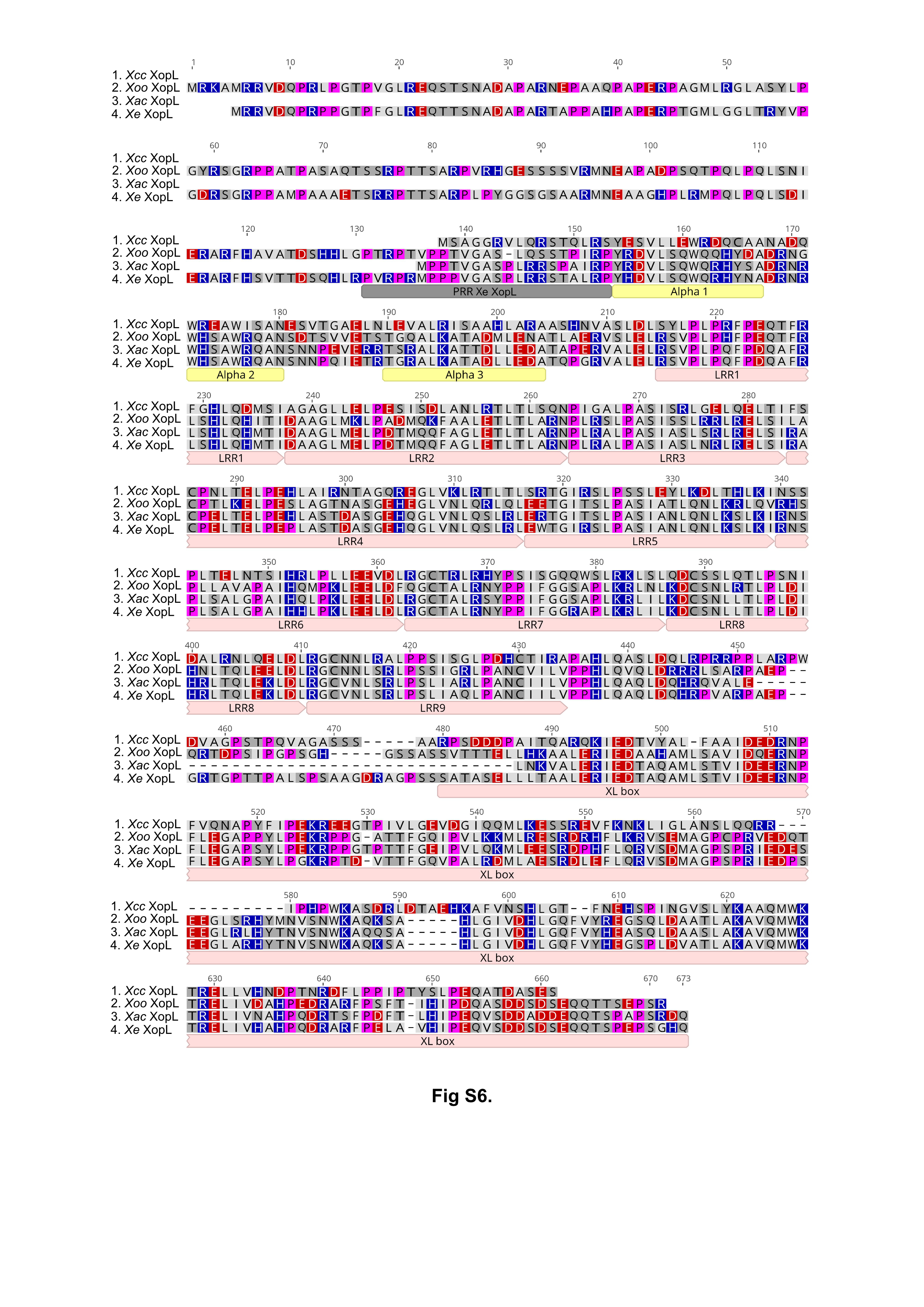

Supplement: S6 Fig — Amino acids are colored based on polarity (Geneious Prime). Acidic amino acids in red, basic in blue, and fuchsia highlights prolines. Domains are marked as follows: the proline-rich region (PRR) in gray, the three alpha helices in yellow, and the 9 LRR repeats and XL box in pink. (TIFF) [file ppat.1011263.s006.tiff]

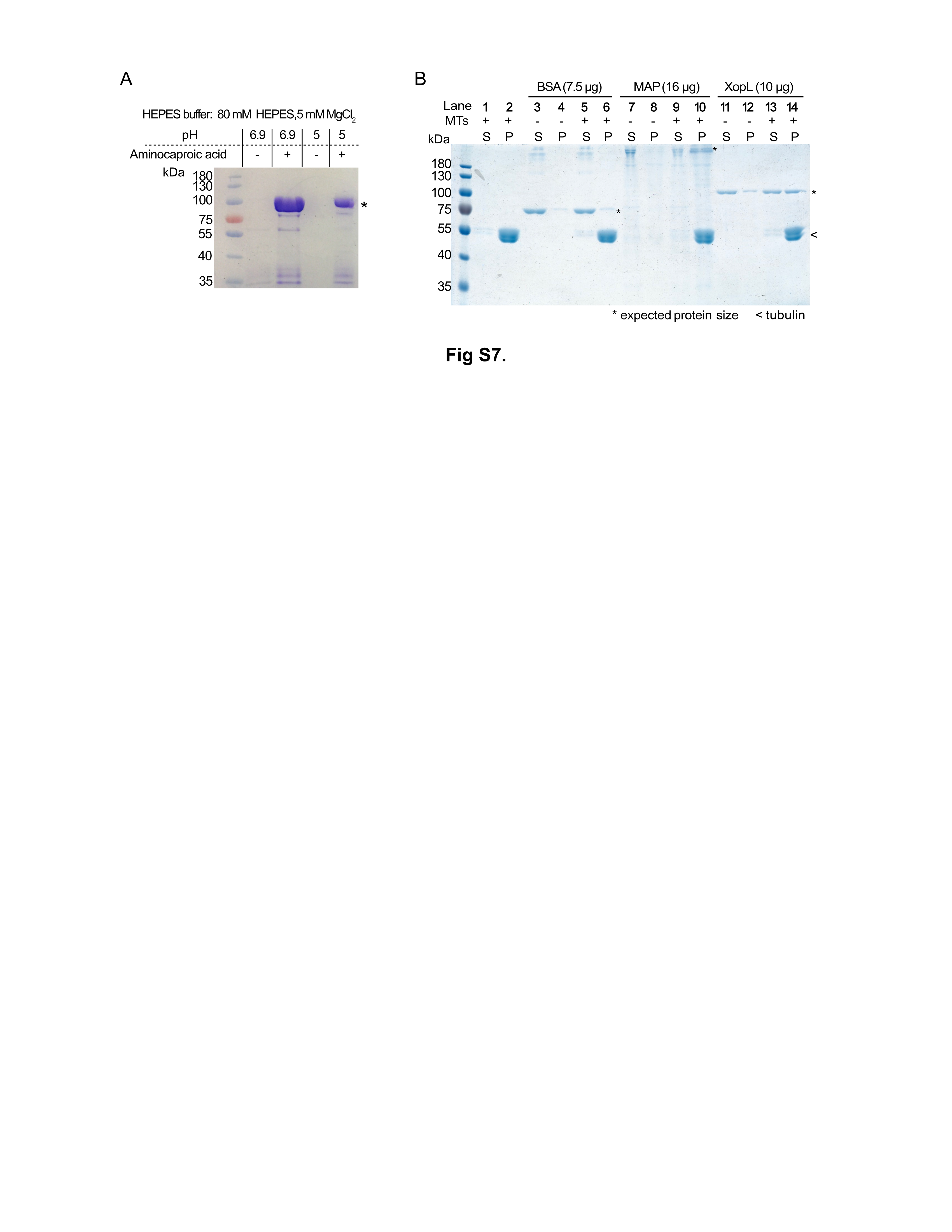

Supplement: S7 Fig — (A) Coomassie-stained 10% polyacrylamide gel showing the optimization of XopLXe solubility under low salt conditions. HEPES buffer without salt at pH 6.9 or 5 was supplemented with 250 mM aminocaproic acid (+) to maximize solubility of 6xHis-StrepII-SUMO-XopL (88.1 kDa) for MT co-sedimentation assays. (B) Coomassie-stained 10% polyacrylamide gel of co-sedimentation assay. Supernatant fractions are marked ‘S’ and pellet fractions ‘P’. Samples with MTs are marked ‘+’ and without by ‘-’. Bands at the expected sizes for the BSA negative control (68 kDa; lanes 3–6), the bovine MAP fraction (a mixture of MT-associated proteins eluted from bovine brain MTs) positive control (250 kDa; lanes 7–10), and XopL (88.1 kDa; lanes 11–14) are marked with ‘*’. A MT only sample served as a control (lanes 1–2). Double tubulin bands marked with ‘<‘. The assay was repeated 2 times with the kit controls, assays with varying XopL concentrations were repeated more than 3 times with comparable results. (TIFF) [file ppat.1011263.s007.tiff]

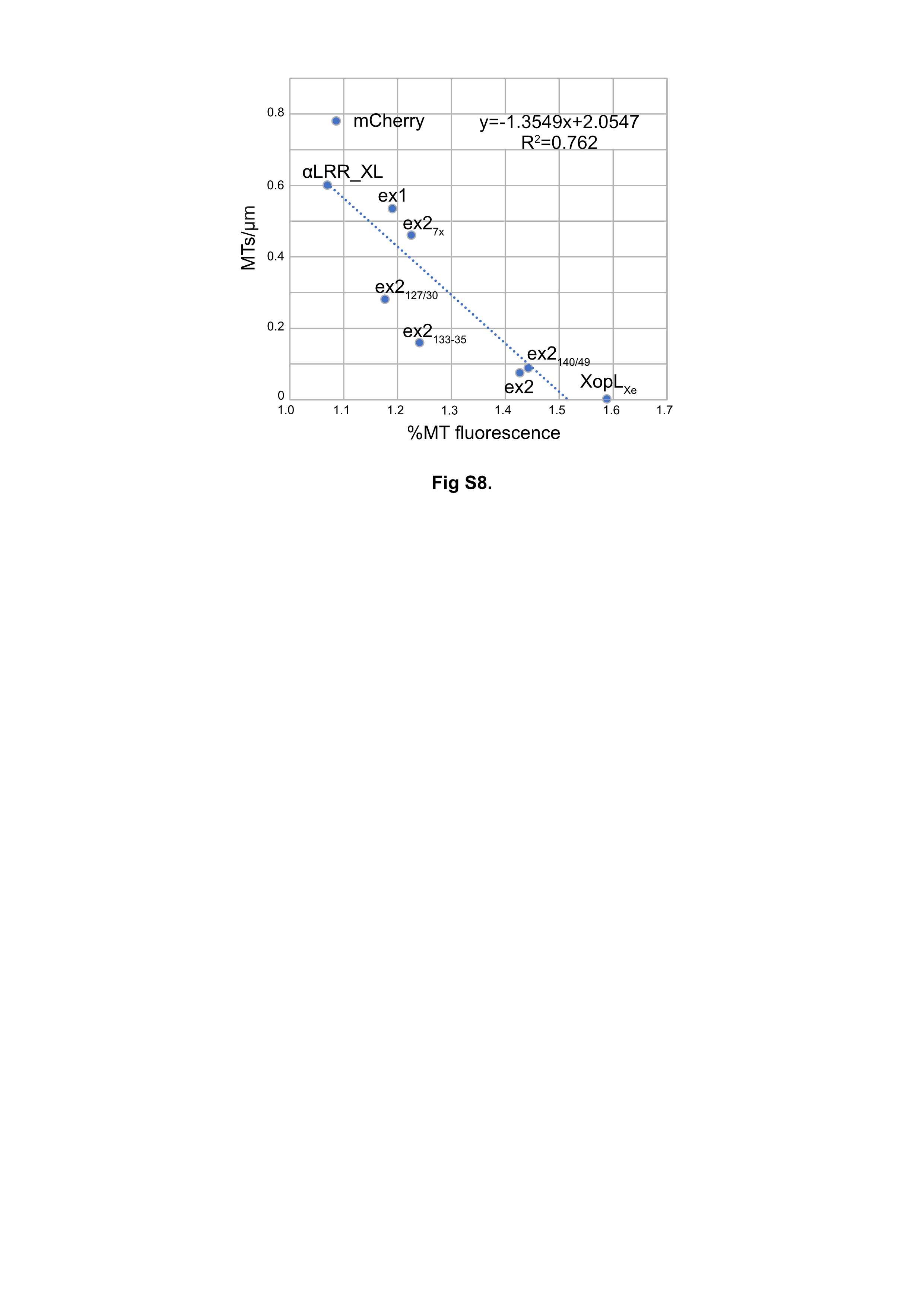

Supplement: S8 Fig — Linear regression comparing MT binding ability of XopLm derivatives with MT number remaining after expression of XopLXe derivatives. Each data point represents the MT association of a given E3 ligase mutant variant (graphed in Fig 5C) plotted against the MT number remaining after expression of the corresponding E3 ligase-active version (graphed in Fig 5D). Each data point is labeled with the derivative name. The line of best fit is blue, and the equation of the line is displayed in the upper right (Linear Regression, R2 = 0.762, F [1, 7] = 22.35, P = 0.002). (TIFF) [file ppat.1011263.s008.tiff]

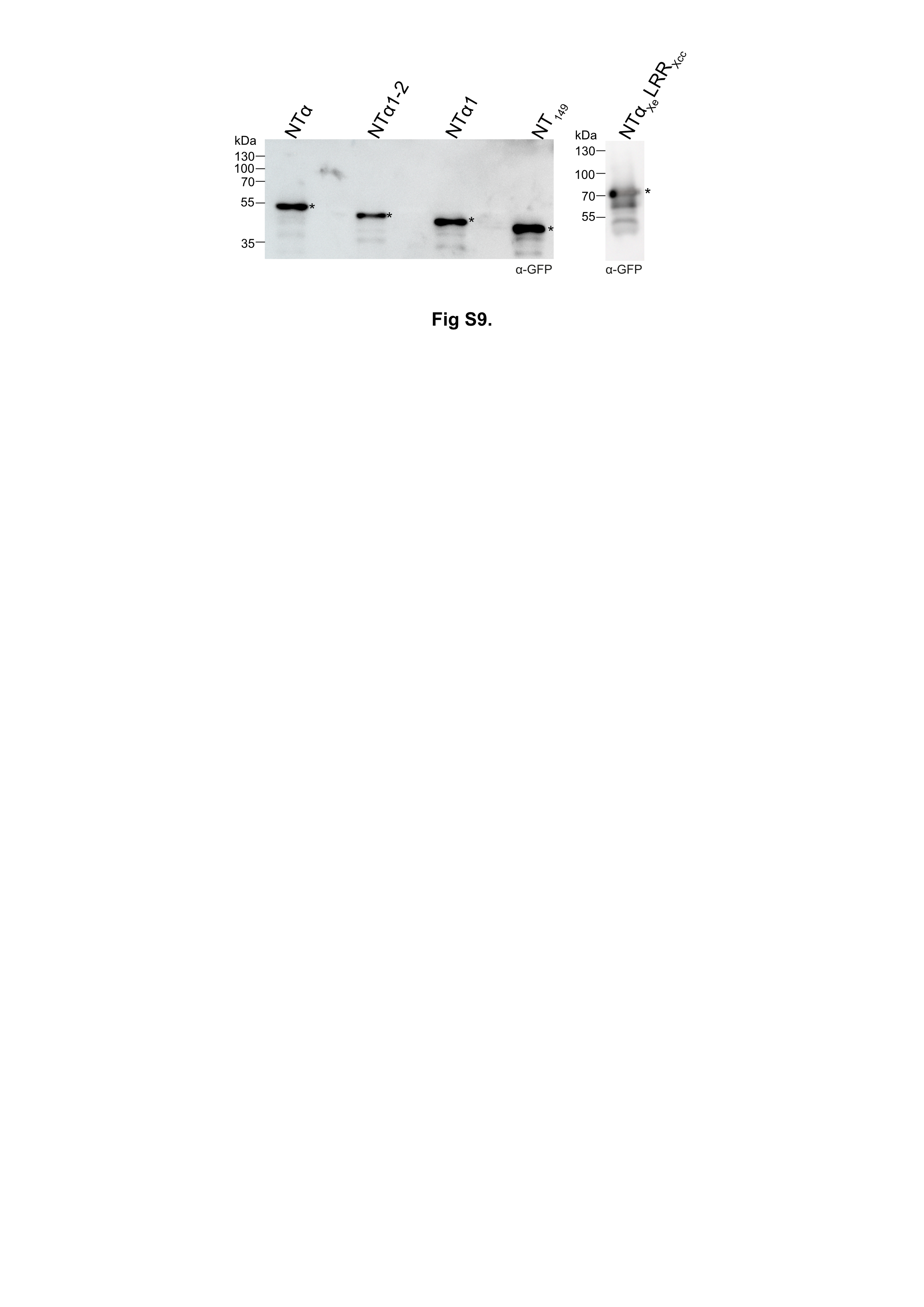

Supplement: S9 Fig — Protein extracts isolated 2 dpi from the experiment depicted in Fig 7. Signals were detected with GFP-specific antibody (*). The left side of the blot shows protein mass in kDa. (TIFF) [file ppat.1011263.s009.tiff]

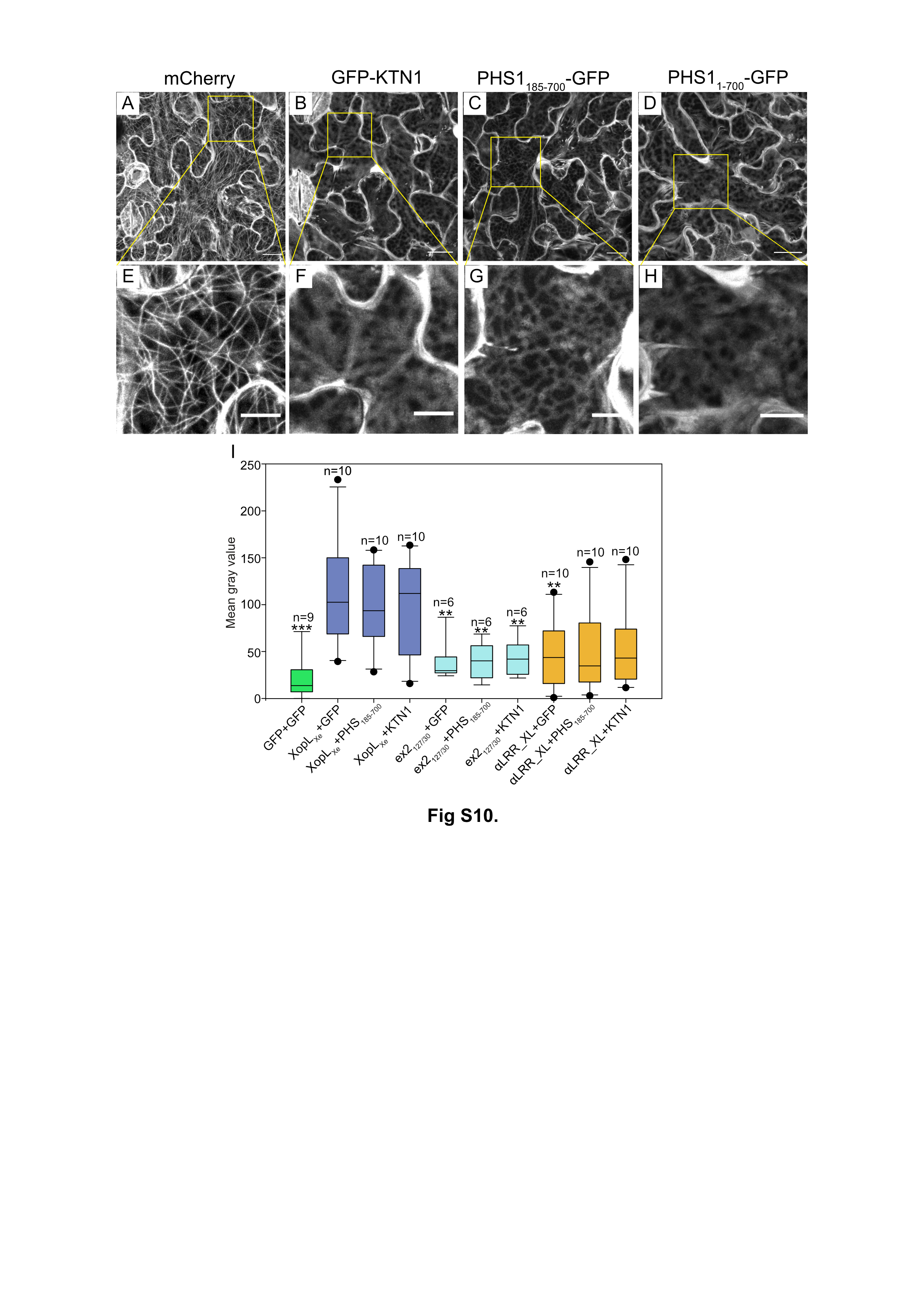

Supplement: S10 Fig — Confocal microscopy of lower epidermal cells of GFP-TUA6 stable transgenic N. benthamiana leaves. Leaves were agroinfected (OD600 of 0.8) to express (A) mCherry, (B) KTN1, (C) PHS1 185-700aa (PHS185-700) or (D) PHS1 1-700aa (PHS1-700) tagged with GFP. Samples were harvested for microscopy at 2 dpi. Images show the GFP channel, where MTs (GFP-TUA6 labeled) are typically visible (i.e., panel A). Scale bars are 20 μm. (E-H) are zoomed in versions of (A-D) respectively. (I) Cell death quantification via red fluorescence scanning of agroinfected N. benthamiana leaves. Tissue co-expressing XopLXe (purple) or non-MT-binding derivatives (ex2127/130; blue and αLRR_XL; orange) together with GFP or MT-disrupting proteins KTN1 and PHS1185-700 was monitored for cell death 5 dpi. Boxes represent first to third quartiles, the median is marked by a horizontal line and whiskers show the distribution of remaining data points. Treatments that were significantly different than XopLXe+ GFP co-inoculations are marked with asterisks (* = p<0.05, ** = p<0.01, *** = p>0.001; One Way Analysis of Variance on Ranks, Bonferroni post-hoc test). (TIFF) [file ppat.1011263.s010.tiff]

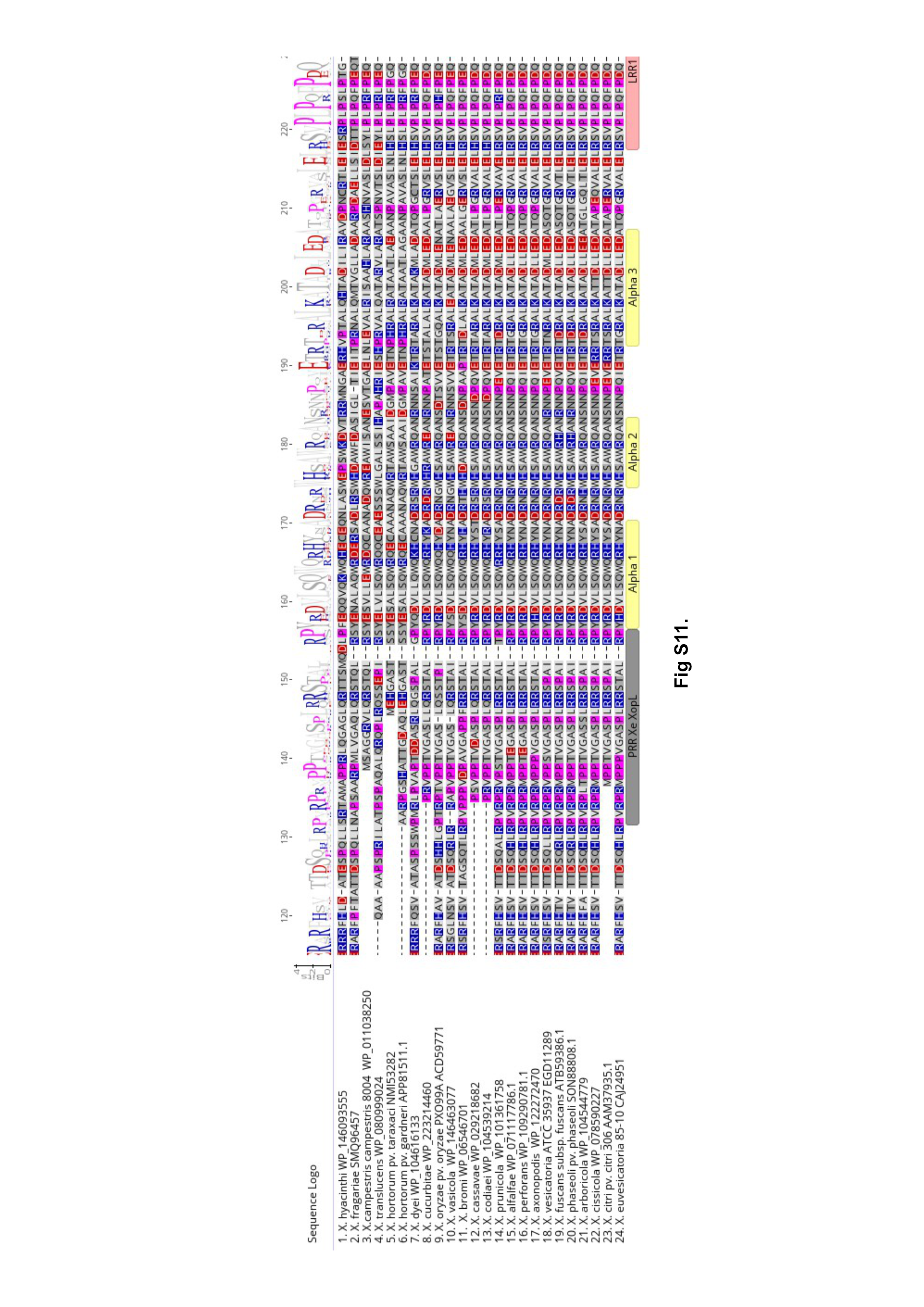

Supplement: S11 Fig — XopL protein sequences from 24 strains were aligned. The strain of origin is listed on the left-hand side with the NCBI accession number of the XopL protein sequence. Amino acids are colored based on polarity (Geneious Prime). Acidic amino acids in red, basic in blue, and fuchsia highlights prolines. The XopLXe proline-rich region (PRR) is in gray, the alpha α region (α-helices 1, 2 and 3) in yellow, and the beginning of the LRR domain (visible as ‘LRR1’) in light pink. The sequence logo above the alignment shows sequence conservation at specific positions. (TIFF) [file ppat.1011263.s011.tiff]
