# Supplementary material for: A conserved microtubule-binding region in Xanthomonas XopL is indispensable for induced plant cell death reactions
Source: PLoS Pathog. 2023 Aug 14;19(8):e1011263. doi: 10.1371/journal.ppat.1011263 (PMC10449215; doi:10.1371/journal.ppat.1011263)
Supplement: Attachment — Submitted filename: 2023 XopL_Reply to Reviewer comments.pdf [file ppat.1011263.s012.pdf]

Response to reviewers

Reviewer text in black and our responses to comments are marked in blue.

Part I - Summary

Please use this section to discuss strengths/weaknesses of study, novelty/significance, general execution and scholarship.

Reviewer #1: In this report the authors used a comparison between polymorphic alleles of the conserved Xanthomonas effector XopL to investigate the role of its subcellular localization in host cell in regard with its functionality.
Using the well-established transient expression system in the model N. benthamiana and live imaging by confocal microscopy, the authors show that several natural variants of XopL can associate with microtubules (MTs) in plant cell and destabilize the MT network. Although XopL appears to directly bind MTs, it does not degrade tubulin, leading the authors to hypothesis that XopL E3-ligase activity must target MT-associated proteins to disturb the MT network. By sequence comparison with the XopLXcc variant that does not associate with MT and using XopL truncations, the authors could identify an N-terminal region of XopL encompassing a proline-rich region (PRR) and a short alpha-helix sufficient for XopL MT-binding. This region bears structural homologies with animals microtubule-associated protein MT-binding domains and is sufficient to confer MT-binding activity to the XopLXcc variant. The importance of the PRR for MT-binding and MT network destabilization was further confirmed by mutagenesis of the proline residues. Lastly, the authors explore the link between XopL MT-binding activity and the induction of cell death, used here as a proxy for XopL function in plants. XopL MT-binding is not sufficient to trigger plant cell death, as the E3-ligase activity is also necessary for this response. The MT network destabilization is not sufficient to induce cell death, as shown by expressing MT network destabilizers, unrelated to XopL. However, XopL-induced cell death correlates with its ability to bind MT when it carries the functional E3 ligase domain, as shown for example with the fusion of the XopLXe-NTa (mediating MT-binding) with the XopLXcc-LRR and XL box domains.
Overall, the study was well designed and conducted with appropriate methods and controls. I commend the authors for the careful interpretation of their data in light of previous studies and the discussion that clearly put in perspectives their findings.

Reviewer #2: This interesting and relevant study does a deep dive into the molecular mechanisms that underpin T3SS effector function in plants. Previously, this group demonstrated that the E3 ligase, XopL, from Xanthamonas spp. associated with the microtubule (MT) cytoskeleton in the absence of E3 ligase activity and may is involved in chloroplast stromule dynamics. Here, by exploring XopL activities, sequence conservation, and domain architecture across Xanthamonas species, they find that XopLxcc from Xanthamonas campestris campestris is unable to bind associate with microtubules. Through deletion analysis, mutagenesis, and domain swap experiments, they demonstrate that MT binding is conferred by a proline-rich tract and the NTα region it is embedded within. Further, the authors show that in planta, microtubules are disrupted by XopLxe and that this perturbation requires E3 ligase activity. Cell death also correlates with the combination of MT-binding and destruction and E3 ligase activity; however, disruption of MTs by overexpression of MAPs is not sufficient to initiate cell death. In a simple pulldown experiment, the authors demonstrate the ability to bind MT in vitro; however, the molecular mechanism of MT destabilization in planta is likely indirect and requires further exploration. I

find this body of work to be impressive, experiments carefully conducted and adequately controlled, and the results critically interpreted.

Reviewer #3: The manuscript （PPATHOGENS-D-23-00407）, “A conserved microtubule-binding region in Xanthomonas XopL is indispensable for induced plant cell death reactions.”, written by Ortmann et al., described their novel finding that the ancestral effector XopL displays bacterial species dependent differences in their sub-cellular localization and plant cell death reactions. It is very interesting that XopL from X. euvesicatoria (XopLXe) directly associates with plant microtubules (MTs) and causes strong cell death in agroinfection assays in N. benthamiana. This event is not associated with XopLXcc which come from X. campestris pv. campestris that fails to localize to MTs and to cause plant cell death. Thus, the authors further confirmed that a proline-rich-region (PRR)/α-helical region is important for MT localization. The MT-localized XopL activity is required for plant cell death reactions. Best to my knowledge, this is the first report that a T3E within the context of a genus rather than a single species can shed light on how effector localization is linked to biochemical activity.
The data are sufficient to support the statement, the manuscript is well organized and written. I would like it accept for publication in this lovely journal.

Major Changes:

Reviewer #1: A main concern (and to generalize the authors findings) is the cell death-inducing activity of XopLXoo and XopLXac. The comparison of XopLXe with these two MT-binding variants could be used to further support the NTalpha region features required for MT-binding (in chimera with Xcc LRR and XL domains) and to clarify the link with cell-death inducing activity of MT-binding XopL variants.

As the reviewer suggested it would provide even more support for the link between microtubule binding and cell death if we could have properly evaluated cell death in $XopL_{Xoo}$ and $XopL_{Xac}$ inoculations. However, since these strains were not available to us, we had the genes synthesized and codon optimized for *N. benthamiana*, but this led to poor/unpredictable expression (Fig 4). Prompted by the reviewers we have sought to optimize the expression and/or stability of these proteins by re-cloning these XopLs with GFP tags (in our experience stability of proteins is sometimes higher when they are tagged with GFP) and tried higher Agrobacterium titers to see if we could create a scenario where expression was better and comparable. We also tried to improve expression with a silencing inhibitor p19. Unfortunately, these approaches did not result in comparable expression between XopL proteins, and, likely as a result of variable expression levels, did not show consistent macroscopic phenotypes when compared to the non-codon optimized versions used for all other experiments. Therefore, we are still not comfortable drawing any conclusions about cell death from this data and have left this aspect of the manuscript as it was. The figure below depicts Westerns as well as leaf pictures that demonstrate variable XopL expression and examples of leaf phenotypes.

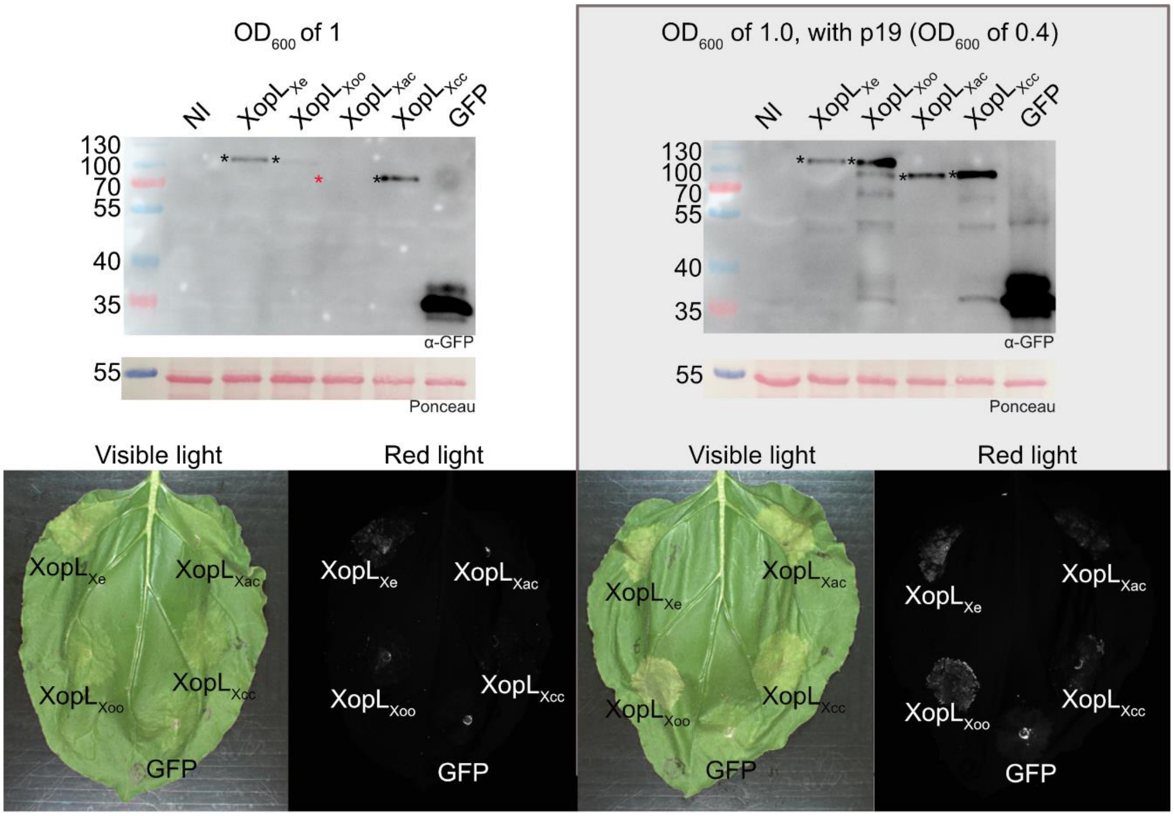

As we mention in the text of the manuscript, we were reluctant to continue with the codon optimized versions of these genes because of this inconsistency. Despite these results, we would like to point out that we have shown sufficient evidence for our claim that the MT-association and cell death are strongly correlated for XopL$_{Xe}$, a finding that is backed by testing of 10 independent XopL variants and quantified in Figure 6 and 8. We would like to acknowledge a mistake we made on p. 20 line 14 and 15 where we wrote:

‘Comparative analyses of the subcellular localization of XopLs after agroinfection revealed that XopL from different *Xanthomonas* species (*Xe*, *Xac* and *Xoo*) associate with microtubules (MTs) *in planta*, triggering their breakdown and ultimately inducing plant cell death.’

This has now been changed to read:

Comparative analyses of the subcellular localization of XopLs after agroinfection revealed that XopL from different *Xanthomonas* species (*Xe*, *Xac* and *Xoo*) associate with microtubules (MTs) *in planta*, ultimately triggering their breakdown. In the case of XopL$_{Xe}$ we were also able to show that MT-association is strongly correlated with plant cell death.

We would like to note that the domain swapping between XopL$_{Xe}$ and XopL$_{Xcc}$ was added as complement to the detailed analysis we did for XopL$_{Xe}$ where we tested amino acid exchanges and truncations extending in either direction to identify the importance of this the NTαLRR for MT-association, and subsequent consequences to cell death caused by the WT protein. Without this level of analysis for the other XopLs (and indeed without knowing for sure whether the WT versions cause cell death in our conditions) we do not want to rely on domain swapping alone to draw conclusions about XopL$_{Xac}$ and XopL$_{Xoo}$.

Reviewer #2: A couple of suggestions for further improvement of these studies:

1) The in vitro microtubule binding studies shown in Fig. 6A should be conducted with a dose series and the data for XopL in the pellet curve fit to estimate a Kd value. Ideally, this would be done with a known MT side-binding protein as a positive control. Finally, binding of the truncated and/or mutated forms of XopL should be examined to demonstrate that the PRR in NTα is necessary and sufficient for MT binding.

The *in vitro* microtubule binding assay was performed to determine if XopL is capable of direct microtubule binding as well as to generally confirm microtubule association in a second context. We find the point raised by the reviewer very interesting and indeed have done a few dose-dependent experiments with XopL to start to investigate the nature of direct binding, which is consistent, but appears to be transient *in vitro* (see figure below). Meaning that there is an equilibrium between MT-bound and unbound XopL protein. However, as you can see and as we mentioned in the manuscript, the low solubility of XopL (as shown by the gray points in the graphs below) presents a significant problem, particularly at higher XopL concentrations.

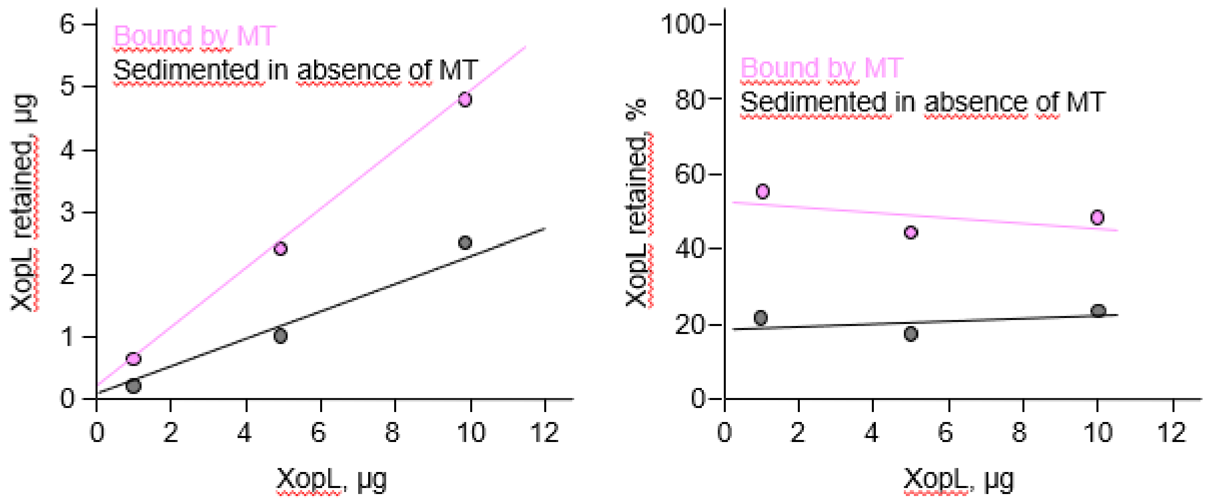

Figure. The left panel shows the total amount of XopL protein added to the *in vitro* MT spin-down assays (x-axis) plotted against the amount retained on the MTs (pink line). The amount of XopL that sediments in the absence of MTs is also plotted (gray). The right panel plots the same data as a percentage of total protein.

While this question is worth exploring, as well as questions related to this, such as how XopL influences microtubule dynamics, and if it binds monomers etc. the focus of this manuscript was primarily on effector-microtubule interactions *in planta*, rather than a detailed biochemical dissection of tubulin binding properties. We hope that the reviewer will understand that to fully address these questions is beyond the scope of this manuscript.

In our eyes current *in vitro* data combined with the MAP-like architecture of XopL provides strong evidence for the direct interaction with MT. As the three reviewers have kindly pointed out, we were careful in our interpretation and description of the *in planta* data. We purposefully employed the term 'microtubule association' whenever possible, in place of 'binding', but we welcome the fact that our data encourage these ideas and see this as confirmation of the relevance of our findings.

2) They have established nice methods to quantify XopL-MT colocalization as well as the effects on microtubule numbers. The former should be applied to the experiments shown in Fig. 7 with the XopL truncations and domain swap constructs to further strengthen the conclusions derived from the observations.

We found this a very reasonable request and we have quantified the association of the different truncations with MTs as suggested (Fig 7H). It nicely complements the microscopy images and further supports our claims. This was a very nice addition to the manuscript. Since the domain swap was performed only to check whether the construct associates with MT we believe that the microscopy image was sufficient to demonstrate this.

I would also suggest to move the excellent correlation analysis shown in Fig. S8 moved into the main text.

We are very happy to hear that Reviewer 2 liked our analysis and we would have liked to include it in the main text. However, since this data is best suited to figure 6, and this figure is quite full, we found it better to include this analysis in the supplement.

Part III – Minor Issues: Editorial and Data Presentation Modifications
Please use this section for editorial suggestions as well as relatively minor modifications of existing data that would enhance clarity.

Reviewer #1: As a general note about the presentation of the results, I recommend the authors to shorten and merge certain parts of the result sections. For example: the 4 XopL alleles E3 ligase activity; the 4 XopL alleles localization; the 4 XopL alleles induced cell-death… Although confocal images are space consuming, splitting controls and samples in different mains and supplemental figures equally splits the attention of readers.

Our rationale for presenting the results in this way and in this order was to tell our story in a series of logical steps that we chose to take based on where the data took us. By merging some of these data for the purposes of compaction we think that the logic behind some of the steps we took would not be clear. I hope the reviewer will forgive us in this case, since we are very satisfied with how the manuscript reads in this order and got positive feedback on this arrangement from Reviewer 3.

We can see the point that some experiments are split between figures. As mentioned by the reviewer, in some cases microscopy pictures take up a great deal of space. At times it was simply not possible to fit all images related to one experiment into a single figure and simultaneously maintain resolution at a level to allow for visualization of fine cellular features like microtubules. However, in case of Fig S1, we have moved this to the main text (into Fig 2) since there was enough space to display the entire experiment there. In general, we tried to divide experiments only when necessary and keep key findings and controls in the main text. I hope this is enough to satisfy the reviewer.

P6l2: hints -> hint (variability and difference in cell death) done

P6l12-22: move to introduction or discussion
We would like to leave this where it is because it is necessary to understand our approach (so it should not be left until the discussion) and yet it does not belong in the introduction because it would dilute our message related to the biological question.

P9l5: rephrase, XopL-fusion expression induced the chloroplast clustering (the XopL-fusion do not "displayed plastid clustering") done

P10l17, p25l23: change "solved" to dissolved in DMSO done

P13l17: Singer et al referencing number? done

P16l9: protein size and accumulation/stability were confirmed done

P16l13: singular (-> XopL-induced decrease in MT abundance is correlated with MT affinity) done

P17l19-20: rephrase "surface-exposed pocket" (?) done

P20l15: XopLXac and XopLXoo did not trigger cell death in benthie (Figure S5) -> see main comment and add reference for Xac and Xoo cell death in benthies/other plants?

We apologize here, the statement made about the cell death was reworded to reflect that the cell death was only correlated with binding for *Xe* XopL. This was simply overlooked in the first version; we were very open about the fact that our codon optimized XopL constructs did not express well and therefore it was not possible to evaluate cell death with confidence.

A reference from XopLXoo-induced cell death in *N. benthamiana* (17) is already mentioned in the introduction.

Figure 1 legend: indicate the expected size of the XopL variants (multiple bands for XopLXe and XopLXoo) Done

Figure 1 UbiGate control: was the XopLm construct tested with this method?
This is a good point, and indeed we used XopLm and multiple truncations to justify our use of UbiGate when we first acquired this system from Marco Trujillo to ensure our results would match previously published data. It is now included as Fig S1.

Figure 2: how is "high" or "low" level of XopLXe-GFP determined? Could the authors indicate the image acquisition conditions (i.e.: gain) used to clarify?
The laser intensities used are now included in the Fig 2 caption. On the Zeiss 780 this is reported as % laser power. This percentage can be a bit misleading however, since the maximum laser power ever used on our microscope is not more than 20-25%. The increase from 10% to 16.8% needed to visualize XopL in the low expressing cells is therefore quite substantial. Gain was kept the same for all images.

Figure 3 legend l3: change "Xopm-mCherry" to XopLm-mCherry done

Figure 3 M: asterisks are not described in the legend and don't appear to be necessary.
A description of the asterisks has been added to the legend.

Figure 3: why not showing TUA6-GFP immunoblot?
A good suggestion, and honestly, this was our original intention, but the TUA6-GFP blots had a lot of background signals, and we were therefore not confident that the TUA signal was discernable. Combined

with the fact that we also wanted to blot for β-tubulin, we then switched to using wild-type plants and blotting for native tubulin proteins.

Figure 4 A and B + legend: the boxes are outlined in black not in white (l6) and difficult to make out.
In our PDF this was not the case, and perhaps this happens during the upload. We will try to make sure this is not the case in the final version.

Figure 6 legend l7-8: replace 3x and 2x by three/ two times done

Figure 6 D: the box plot for XopLXe is not on the graph…
$XopL_{Xe}$ actually was there, but indeed, it was not very visible. XopL treatments usually result in values of 0 MT/µm. We have made this line thicker so there is no more confusion here.

Figure 6 E: no loading control?
Yes, there was no loading control included. The signal of the CTD is around the size of rubisco and this signal was still visible on the membrane after development and was apparent in the amido black stain. For this reason, we chose not include the loading control. Since we did not want to make any comparisons of protein level between the different samples and only used the western to show the proteins are present and at the right size we thought this was appropriate. However, we are fine with including it at the reviewers request and have now done so.

Figure 8 C: this panel should be split into experiment shown in A and experiment shown in B as these were done independently. done

Figure S6 and S11: change Xcv to Xe for the PRR box
We would like to thank the reviewer for catching this. It has now been corrected.

Figure S10 I: add y-axis title (Cell death or mean gray value)
We would like to thank the reviewer for catching this. It has now been corrected.

Reviewer #2: N/A

Reviewer #3: There are no minor issues for revision.
